# Cost-effectiveness of biomarker-directed toripalimab plus chemotherapy for previously untreated extensive-stage small-cell lung-cancer in China

**DOI:** 10.1371/journal.pone.0328730

**Published:** 2025-07-24

**Authors:** Shuo Kang, Shan Zhao, Xiaohui Wang, Zhenhua Pan

**Affiliations:** 1 Medical Insurance Office, The Second Hospital of Hebei Medical University, Shijiazhuang, Hebei Province, People's Republic of China; 2 Department of Oncology, The Second Hospital of Hebei Medical University, Shijiazhuang, Hebei Province, People's Republic of China; 3 Development Planning Division, Hebei Medical University, Shijiazhuang, Hebei Province, People's Republic of China; Sun Yat-Sen University, CHINA

## Abstract

**Background:**

With or without biomarker-directed toripalimab plus chemotherapy could bring significant clinical benefit and acceptable safety profile compared with chemotherapy as first-line treatment for patients with extensive-stage small-cell lung-cancer (ES-SCLC) were demonstrated in EXTENTORCH trial. However, its cost-effective remains uncleared.

**Objective:**

The current analysis aimed to evaluate the economic value of intratumor heterogeneity (ITH) testing directed toripalimab plus chemotherapy as first-line treatment for patients with ES-SCLC from the Chinese health-care system perspective.

**Design:**

A mathematical decision model-based cost-effectiveness analysis,

**Methods:**

A pharmacoeconomic decision model was developed to simulate 3-week patients transition in 20-year time horizon to access the cost-effectiveness of three competing first-line treatments among ITH-testing directed toripalimab plus chemotherapy, toripalimab plus chemotherapy, and chemotherapy alone. Survival data were obtained from EXTENTORCH trial, cost and utility values were gathered from the dataset and published studies, annual discount rate of 5% was used for cost and utility values. Total cost, life-years (LYs), quality-adjusted life-years (QALYs), and incremental cost-effectiveness ratio (ICER) were the model outputs. One-way and probabilistic sensitivity analyses were conducted to estimate the robustness of the model results,

**Results:**

In base-case analysis, compared with toripalimab plus chemotherapy and chemotherapy alone, ITH-testing directed therapy could bring additional 0.14 QALYs and 0.29 QALYs, with marginal costs of $3750.75 and $7778.18, resulting in the ICER of $27,353.27/QALY and $26,461.46/QALY, respectively, which lower than the Chinese willingness-to-pay (WTP) threshold. Sensitivity analyses demonstrated the model results were robust, probabilistic sensitivity analyses showed the probability of ITH-testing directed therapy could be considered cost-effective was 61%.

**Conclusions:**

ITH-testing directed treatment was likely to be the most cost-effective first-line option compared with toripalimab plus chemotherapy and chemotherapy alone for patients with previously untreated ES-SCLC from the Chinese health-care system perspective.

## Introduction

Lung cancer has the highest incidence and mortality of all malignancy worldwide [1]. Small-cell lung-cancer (SCLC) is the most aggressive subtype of all lung cancer according to the histological type. Unfortunately, approximately 66% of SCLC patient had progressed to the extensive-stage SCLC (ES-SCLC) with metastasis at the initial diagnosis [2,3], which with the especially poor prognosis and 5-year survival rate was less than 7% [4]. The chemotherapy of platinum plus etoposide was the standard first-line treatment over past 30 years which achieved good initial results but rapidly progressed and resulting the limited median overall survival was only 10 months [5–7].

Recently, immune checkpoint inhibitors (ICIs) could reactive the anti-tumor function of T cells by inhibiting the programmed cell death-1 (PD-1) and programmed cell death receptor ligand-1 (PD-L1) pathway [8–10]. The addition of ICIs such as atezolizumab, durvalumab, and serplulimab to first-line platinum plus etoposide demonstrated the clinical and safety profile compared with chemotherapy alone [11–13]. Toripalimab, is a novel immunoglobulinG4PD-1 blocking antibody, the recent EXTENTORCH trial demonstrated that toripalimab plus chemotherapy could significantly reduce the risk of disease progression or death by 33% (hazard ratio (HR), 0.67, 95% confidence interval: 0.54–0.82) and the risk of death by 20% (HR, 0.80, 95% confidence interval: 0.65–0.98) for untreated ES-SCLC patients compared with chemotherapy alone. Simultaneously, EXTENTORCH trial found that the biomarker of intratumor heterogeneity (ITH) layered selected therapy could further enhance the clinical value of toripalimab plus chemotherapy compared with chemotherapy alone, which could prolong the median progression-free survival (PFS) and median overall survival (OS) for ITH-low patient [14].

Despite toripalimab plus chemotherapy demonstrated clinical benefit compared with chemotherapy alone, we need pay more attention to its cost-effective benefits with the widely used in the clinical practice in resource-limited countries such as China, although the price of toripalimab has significantly decreased since its initial launch, it was still relatively expensive compared with chemotherapy. The aim of the current analysis was to evaluate the cost-effectiveness of adding toripalimab to first-line chemotherapy for patients with previously untreated ES-SCLC from the Chinese health-care system perspective.

## Methods

### Analytical overview and model structure

**Fig 1:**
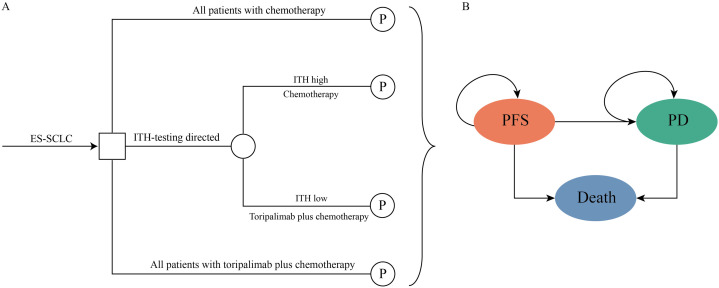
The structure of the mathematical decision model. ES-SCLC, extensive-stage small-cell lung-cancer; ITH, intratumor heterogeneity; PFS, progression-free survival; PD, progressed disease.

A mathematical model combining the decision tree process and partitioned survival model (PSM) was established to evaluate the cost-effectiveness of biomarker-directed toripalimab plus chemotherapy for patients with untreated ES-SCLC. The decision tree displayed a clear process of the decision-making of the three strategies of first-line therapy of ES-SCLC (Fig 1A), among patients used chemotherapy or toripalimab plus chemotherapy without biomarker detection, and biomarker-directed strategy, which patients with ITH low or high used toripalimab plus chemotherapy or chemotherapy alone, respectively. The PSM was developed to simulate the disease course of the ES-SCLC in the hypothetical cohort (Fig 1B), the PSM was constituted by three mutually exclusive health states: PFS, progressed disease (PD), and death. All patients were at PFS state when entered the model, and the patients could not return to the previous health states. The PSM simulated every 3-week patients transition in 20-year time horizon. Patients would progress to another health state or keep at the current health state at the end of each cycle.

Total costs, life-years (LYs), quality-adjusted life-years (QALYs) were the model outputs, costs and utility values which used to estimate the QALYs were discounted at an annual rate of 5% according to the Chinese guidelines for pharmacoeconomic evaluations [15]. Costs values were shown in 2023 US dollars (US $1 = CNY 7.047), incremental cost-effectiveness ratio (ICER) was calculated by the following formula: ICER=(C_1_-C_2_)/(E_1_-E_2_), which presented as costs per additional QALY gained to judge the cost-effective of competing first-line treatment. Three times of per capita gross domestic product (GDP) of China in 2024 ($40,761/QALY) was set to be the WTP threshold in line with the WHO recommendations [16–18]. This research did not involve any human or animal subjects, thus ethical approval and consent to participate were not required.

### Clinical data

The clinical survival and safety data were gathered from the EXTENTORCH trial. Individual time-to-event data were reconstructed by using the standard algorithm developed by Guyot et al [19], then the individual patient data were used to fit the following parametric survival model among, Exponential, Gamma, Generalized Gamma, Weibull, Log-normal, Log-logistic, Gompertz [20]. Goodness between the fitting survival model and the Kaplan-Meier (K-M) curves reported in the EXTENTORCH trial was based on the visual inspection and Akaike information criterion (AIC), AIC values and best fitted models were performed in Supplementary Table 1. The estimated survival parameters were shown in Table 1. After the disease progressed, patients would receive the standard second-line treatment of topotecan, and the proportion of patients received second-line therapy in the three strategies were gathered from the EXTENTORCH trial.

**Table 1:** Survival model parameters fitting to the PFS and OS data from the EXTENTORCH trial.

Treatment	PFS		OS	
	Model	Parameters	Model	Parameters
Toripalimab plus chemotherapy	Log-logistic	Shape = 2.276; Scale = 6.265	Log-logistic	Shape = 2.185; Scale = 15.173
Chemotherapy	Log-logistic	Shape = 3.340; Scale = 5.417	Log-logistic	Shape = 2.335; Scale = 13.126
ITH-testing directed toripalimab plus chemotherapy	Log-logistic	Shape = 1.700; Scale = 7.631	Log-logistic	Shape = 1.586; Scale = 21.149
ITH-testing directed chemotherapy	Weibull	Shape = 2.176; Scale = 6.124	Weibull	Shape = 1.78; Scale = 17.41

PFS, progression-free survival; OS, overall survival; ITH, intratumor heterogeneity.

### Cost and utility values

Only direct medical costs were considered in the current analysis because the evaluate perspective was the Chinese health-care system, costs values included the cost of anti-cancer regimens, routine follow-up, best supportive care, end-of-life care, and management of treatment-related serious adverse events (SAEs, grade≥3), typical patient with weight of 65 kg and height of 1.64m, resulting in the body surface area (BSA) of 1.72m^2^ [21], was used to calculate the dosage of the chemotherapy, all cost values were obtained from the local bid-winning price or previously literatures.

The health utility values which reflecting health state preference that affected by race, region, and religious beliefs were obtained from the related studies and the utility values were usually on the scale of 0–1, the utility values of the PFS, PD, and Death health state in the current study were set to be 0.673, 0.473 and 0 [22], respectively, the disutility values caused by the SAEs were also considered in our model. All model parameters were shown in Table 2.

**Table 2:** Model inputs: base-line values and ranges for sensitivity analyses.

Parameter	Base-line value	Range		Reference
		Lower bound	Upper bound	
Cost values (US $)				
Toripalimab per 240mg	267.47	200.60	334.34	Local-charge
Carboplatin per 100mg	10.7	8.025	13.375	Local-charge
Cisplatin per 10mg	2.5	1.875	3.125	Local-charge
Etoposide per 100mg	37.95	28.05	47.44	Local-charge
Topotecan per 1mg	26.73	20.05	33.41	Local-charge
Routine follow-up per cycle	73.9	59.1	88.7	[23]
Best supportive care per cycle	359.524	269.643	449.405	[24]
End-of-life care	2221	1777	2665	[25]
ITH testing	2128.57	1596.43	2660.71	Local-charge
Decreased WBC count per event	466	372.8	559.2	[26]
Decreased platelet count per event	1054	843.2	1264.8	[26]
Anemia per event	508.2	406.56	609.84	[27]
Utility and disutility values				
PFS	0.673	0.538	0.808	[22]
PD	0.473	0.378	0.568	[22]
Decreased WBC count	-0.2	-0.24	-0.16	[28]
Anemia	-0.073	-0.088	-0.058	[28]
Decreased platelet count	-0.19	-0.23	-0.15	[28]
Risk of SAEs in toripalimab plus chemotherapy group				
Decreased WBC count	0.387	0.323	0.451	[14]
Anemia	0.306	0.245	0.367	[14]
Decreased platelet count	0.248	0.191	0.305	[14]
Risk of SAEs in chemotherapy group				
Decreased WBC count	0.449	0.383	0.515	[14]
Anemia	0.347	0.284	0.410	[14]
Decreased platelet count	0.343	0.280	0.406	[14]
Others				
proportion of patients received carboplatin in toripalimab plus chemotherapy group	0.713	0.654	0.772	[14]
proportion of patients received carboplatin in chemotherapy group	0.731	0.672	0.789	[14]
proportion of patients received second-line topotecan in toripalimab plus chemotherapy group	0.552	0.487	0.617	[14]
proportion of patients received second-line topotecan in chemotherapy group	0.694	0.633	0.755	[14]
Discount rate	0.05	0	0.08	[15]

ITH, intratumor heterogeneity; WBC, white blood cell; PFS, progression free survival; PD, progressed disease.

### Sensitivity analyses

Sensitivity analyses included one-way and probabilistic sensitivity analyses (PSA) were conducted to evaluate the robustness of the model results when model parameters were changed. In one-way sensitivity analyses, parameters were changed one-by-one based on their lower and upper bound to identify which was the most influence parameter of the model results, the range of the parameter was set as 95% confidence interval or ±25% of the base-line value (when the 95% confidence interval was not available). The results of the one-way sensitivity analyses were presented by the Tornado diagram. For PSA, Monte Carlo simulation of 1,000 iterations was developed by repeatedly sampling the model parameters from the statistical distributions, where Gamma distribution was used for cost values, Beta distribution was used for utility and disutility values, incidence rates proportions and probabilities [29], the results of PSA were presented by the cost-effectiveness acceptability curves (CEACs).

## Results

### Base-case results

From Chinese health-care system perspective, in 20-year time horizon, ITH testing directed treatment could bring additional 0.14 QALYs and 0.29 QALYs with the acceptable marginal costs of $3750.75 and $7778.18 compared with toripalimab plus chemotherapy and chemotherapy alone, resulting in the ICER of $27,353.27/QALY and $26,461.46/QALY, respectively, which were lower than the Chinese WTP threshold of $40,761.60 per additional QALY gained, the base-case results were shown in Table 3.

**Table 3:** Base-case results.

Strategy	Costs ($)	LYs	QALYs	ICER ($/QALY)
Chemotherapy	16,617.13	1.52	0.77	26,461.46
Toripalimab plus chemotherapy	20,644.56	1.83	0.92	27,353.27
ITH-testing directed treatment	24,395.31	2.22	1.06	–

LYs, life-years; QALYs, quality-adjusted life-years; ICER, incremental cost-effectiveness ratio; ITH, intratumor heterogeneity.

### Sensitivity analyses results

**Fig 2:**
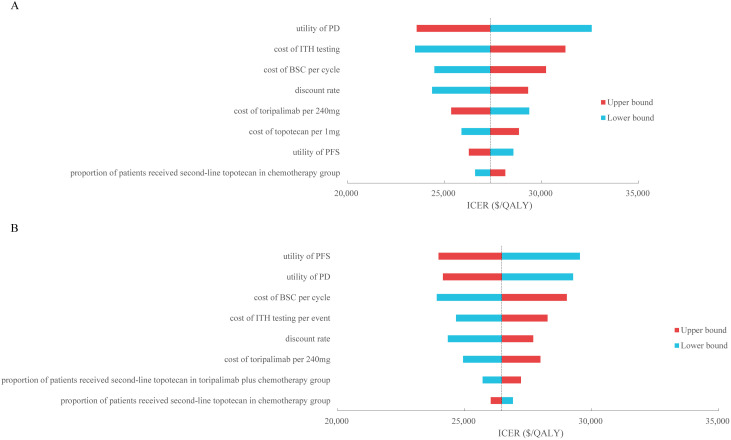
Tornado diagram of one-way sensitivity analyses of (A) ITH-testing directed treatment versus toripalimab plus chemotherapy and (B) ITH-testing directed treatment versus chemotherapy. PFS, progression-free survival; PD, progressed disease; ITH, intratumor heterogeneity; BSC, best supportive care; ICER, incremental cost-effectiveness ratio; QALY, quality-adjusted life-year.

In one-way sensitivity analyses, for ITH-testing directed treatment versus toripalimab plus chemotherapy, the utility of PD was the main driver of the model outcomes (Fig 2A), for ITH-testing directed treatment versus chemotherapy alone, the utility of PFS had the substantial influence of the model results (Fig 2B). For the two comparisons, the ICERs were always lower than the WTP threshold of $40,761/QALY whatever each parameter changed in the threshold range, the results of one-way sensitivity analyses were robustness.

**Fig 3:**
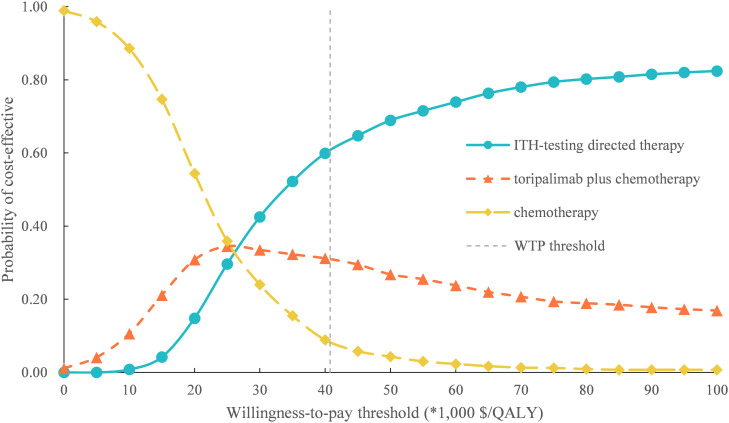
Cost-effectiveness acceptability curves of three competing first-line therapy. ITH, intratumor heterogeneity; WTP, willingness-to-pay; QALY, quality-adjusted life-year.

For PSA, the probability of ITH-testing directed treatment, toripalimab plus chemotherapy, and chemotherapy alone could be considered cost-effective at the Chinese WTP threshold of $40,761 per additional QALY gained was 61%, 31%, and 8%, respectively (Fig 3). These findings suggested the ITH-testing directed treatment was the most cost-effective first-line therapy but sensitive to utility assumptions for patients with previously untreated ES-SCLC. We also conducted additional exploratory analysis on the model of the price reduce of toripalimab, we found that when the price of toripalimab reduced 30% and 50%, the cost-effective probability of ITH-testing directed treatment was 60%, and 58%, respective, at the WTP threshold of $40,761/QALY (Supplementary Fig 1 and Supplementary Fig 2).

## Discussion

Both oncologists and health-care decision makers were interested by the results of the clinical benefit of adding toripalimab to first-line chemotherapy for patients with previously untreated ES-SCLC in EXTENTORCH trial, simultaneously, ITH-testing results layered treatment option of toripalimab plus chemotherapy demonstrated superior clinical benefit compared to it in the intention-to-treat population. Although the price of toripalimab has been significant decline compared with initial launch, its price was still relatively expensive compared to chemotherapy, and its cost-effective was need to be clarified. The current analysis aimed to evaluate the cost-effectiveness of biomarker-directed toripalimab plus chemotherapy as first-line treatment for patients with ES-SCLC, the study has three competing therapies among all patients with toripalimab plus chemotherapy, all patients with chemotherapy, and patients with ITH-testing results directed treatment. In base-case analysis, ITH-testing directed treatment could bring additional health benefit compared with toripalimab plus chemotherapy and chemotherapy alone with the acceptable marginal cost, resulting in the ICER of $27,353.27/QALY, and $26,461.46/QALY, respectively, which lower than the WTP threshold of $40,761 per additional QALY gained, one-way sensitivity analyses suggested that the utility value of the health state was the main driver of the model results, PSA showed that the probability of ITH-testing directed treatment could be considered cost-effective was 61%, and to explore additional contexts, we conducted the price analysis, we found that when the price of toripalimab reduced 30% and 50%, the cost-effective probability of ITH-testing directed treatment was stabled at around 60%, these findings revealed that the ITH-testing directed treatment was likely to be the most cost-effective first-line treatment but sensitive to utility assumptions for patients with previously untreated ES-SCLC.

Notably, in the current analysis, the ITH testing method was Mutant-Allele Tumor Heterogeneity (MATH) method, which is a computational tool designed to quantify intratumor heterogeneity by analyzing the dispersion of variant allele frequencies (VAFs) across somatic mutation loci within tumor samples using high-throughput sequencing data, and the score of 29 was used as the boundary between high and low heterogeneity. In China, biomarker-informed precision decision-making was received growing emphasis in the management of malignant neoplastic diseases, and with widespread application in the clinical practice, it was critical for enhancing rational drug utilization and optimizing healthcare resource allocation efficiency. Our analysis conducted the economic evaluation based on potential clinical pathways, wherein the cost of ITH testing was gathered from the local charge, and the ITH testing method of MATH was feasibility for the clinical practice, the results of our analysis demonstrated the economic value of refined clinical pathway, which was valuable for health-care decision makers.

Several studies evaluated the cost-effectiveness of adding ICIs to first-line chemotherapy compared chemotherapy alone from the Chinese context, the analyses demonstrated that adding atezolizumab, durvalumab, or adebrelimab was unlikely to be the cost-effective first-line treatment due to the unfavorable ICERs with limited health benefit and unacceptable marginal cost [30–32], these finds were in line with the understanding with the lower economic conversion rate of the high-price innovative anti-cancer drug. The above issues were still the direction of health-care decision makers need to pay more attention to. Although the development of Chinese economy and decreased of the drug price, the cost-effective of the high-value treatment may be improved, the future potential market imbalance need to be monitored. As the innovation point of the current study, we also involved the ITH-testing directed treatment in our analysis, and we found that it was more cost-effective than the competing strategies which were not considered biomarkers for using toripalimab plus chemotherapy or chemotherapy alone, our findings were valuable for treatment decision based on the precise economic evaluation results for both oncologists and health-care decision makers.

Several limitations must be illustrated when the health-care decision making was based on our study results. First, long-time survival data beyond the follow-up period in the clinical trial which used to calculate the health benefit were extrapolated by fitting the parametric survival model, despite the potential bias may be caused between the model results and real-world data, this method was an inevitable limitation of the cost-effectiveness analysis for anti-cancer treatment. Second, some cost values such as the cost of routine follow-up, the cost of best supportive care were gathered from the published studies rather than real-world data, however, sensitivity analyses demonstrated the robustness of the model outputs when parameters were changed. Only direct medical costs were considered in the model due to the health-care system perspective was conducted in the current analysis, however, as the chronic disease management model for malignant tumor diseases continues to deepen and refine, with more rational measurement methods become widespread, indirect costs such as productivity loss expenses caused by the disease should be increasingly considered for inclusion in such research. Third, as the key parameter for evaluating the health benefit, health state utility values, which may be affected by ethnicity and religious were obtained from a foreign study, although the utility values were the main driver of the model results, one-way sensitivity revealed that the ICERs were always lower than the WTP threshold, the model results were robustness. Fourth, to ensure the simplicity of the model structure, the cost of management of grade 1/2 adverse events were excluded from the model, only the cost of management SAEs (Grade≥3) was considered in the model, however, only minimal influence of the model outcomes for these parameters in our analysis. Finally, other potential competing first-line ICIs plus chemotherapy treatment for patients with ES-SCLC were not included in the current analysis due to the absence of the head-to-head clinical study. Despite the above limitations, we believe our analysis findings reflected the clinical and economic outcomes of the three first-line treatments for patients with previously untreated ES-SCLC in Chinese context.

## Conclusion

In conclusion, ITH-testing directed therapy could be considered the most cost-effective first-line option compared with toripalimab plus chemotherapy and chemotherapy alone for patients with previously untreated ES-SCLC from the Chinese health-care system perspective due to the acceptable ICER.

## Supporting Information

S1 Table Summary of statistical goodness-of-fit of Kaplan-Meier curves in EXTENTORCH trial. (PDF)

S1 Fig Cost-effectiveness acceptability curves of three competing first-line therapy when the price of toripalimab reduced 30%. ITH, intratumor heterogeneity; WTP, willingness-to-pay; QALY, quality-adjusted life-year.
(TIF)

S2 Fig Cost-effectiveness acceptability curves of three competing first-line therapy when the price of toripalimab reduced 50%. ITH, intratumor heterogeneity; WTP, willingness-to-pay; QALY, quality-adjusted life-year.
(TIF)
