# Supplementary material for: Cost-effectiveness of biomarker-directed toripalimab plus chemotherapy for previously untreated extensive-stage small-cell lung-cancer in China
Source: PLoS One. 2025 Jul 24;20(7):e0328730. doi: 10.1371/journal.pone.0328730 (PMC12288987; doi:10.1371/journal.pone.0328730)
Supplement: S1 Table — (PDF) [file pone.0328730.s001.pdf]

Supplementary Table 1 Summary of statistical goodness-of-fit of Kaplan-Meier curves in EXTENTORCH trial.

	AIC			
	Toripalimab plus chemotherapy	Chemotherapy	ITH-testing directed treatment of toripalimab plus chemotherapy	ITH-testing directed treatment of chemotherapy
PFS				
Exponential	1072.593	1094.813	299.1632	332.9644
Gamma	1031.457	963.7735	297.773	294.0409
Generalized-Gamma	1026.712	964.6701	298.2552	294.0502
Weibull	1041.928	968.6719	298.842	292.3737
Log-normal	1026.889	988.0335	297.159	303.6645
Log-logistic	1013.28	958.8101	293.7745	296.4489
Gompertz	1068.524	1015.947	301.1604	305.2884
OS				
Exponential	1399.956	1432.377	357.5891	420.822
Gamma	1358.447	1379.849	357.1561	401.6022
Generalized-Gamma	1359.542	1381.833	359.1318	402.4613
Weibull	1363.873	1383.972	357.1886	400.4866
Log-normal	1368.308	1402.466	365.0215	412.558
Log-logistic	1349.148	1373.95	356.0039	403.7163
Gompertz	1386.323	1407.274	358.8709	405.5557

AIC Akaike information criterion, *ITH* intratumor heterogeneity, *PFS*, progression-free survival, *OS* overall survival.
