# Supplementary figures and images for: Cost-effectiveness of biomarker-directed toripalimab plus chemotherapy for previously untreated extensive-stage small-cell lung-cancer in China
Source: PLoS One. 2025 Jul 24;20(7):e0328730. doi: 10.1371/journal.pone.0328730 (PMC12288987; doi:10.1371/journal.pone.0328730)

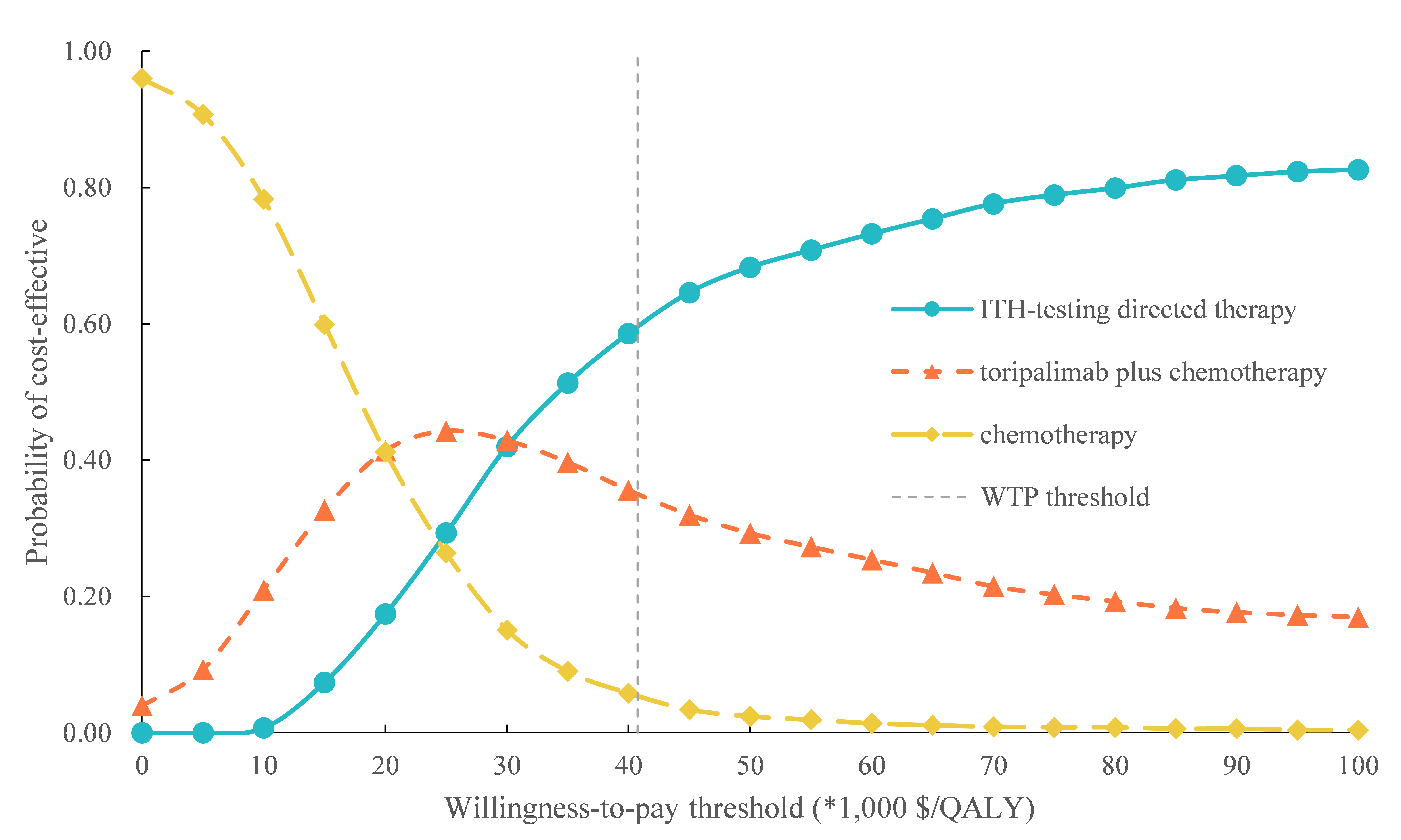

Supplement: S1 Fig — ITH, intratumor heterogeneity; WTP, willingness-to-pay; QALY, quality-adjusted life-year. (TIF) [file pone.0328730.s002.tif]

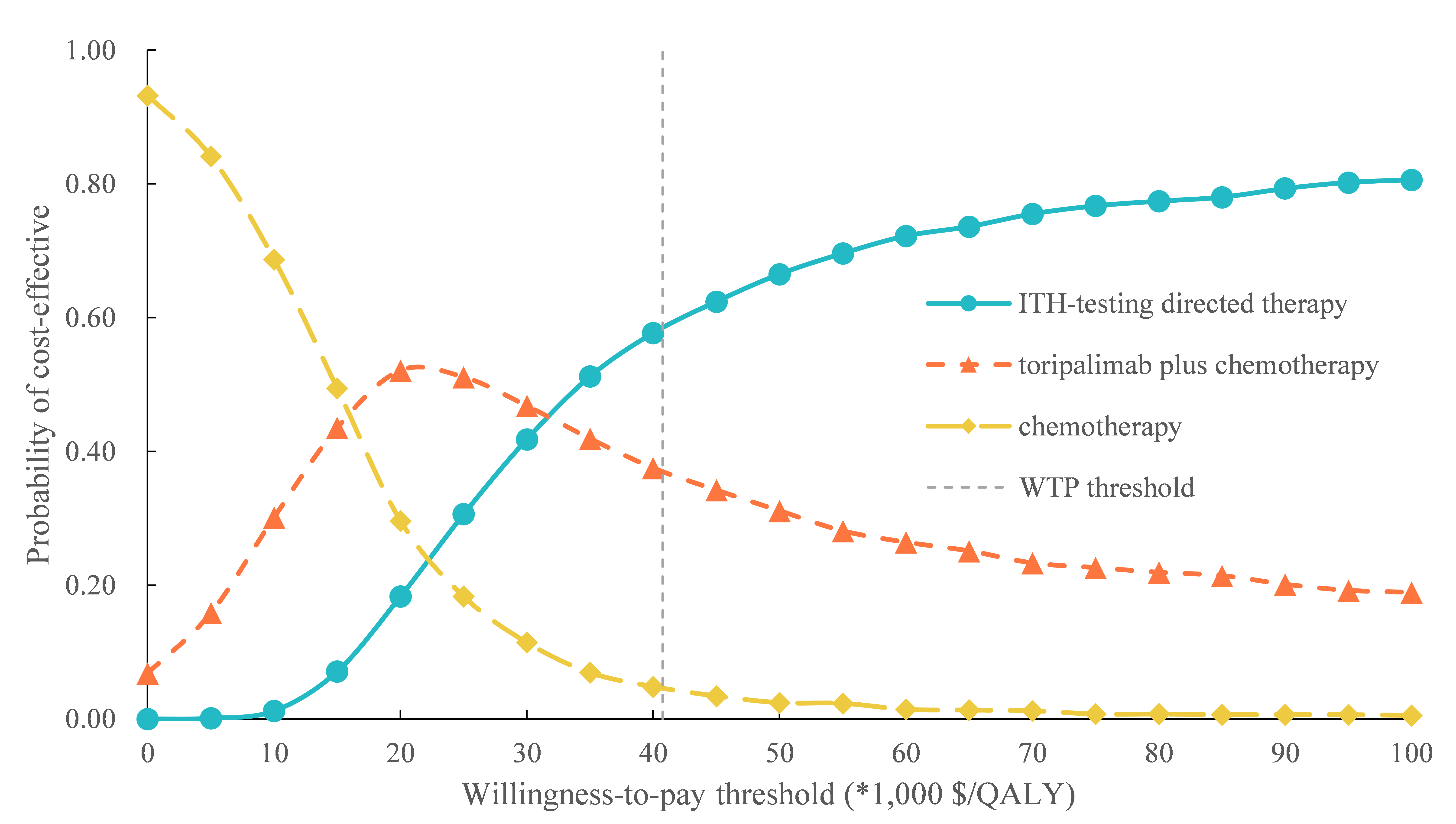

Supplement: S2 Fig — ITH, intratumor heterogeneity; WTP, willingness-to-pay; QALY, quality-adjusted life-year. (TIF) [file pone.0328730.s003.tif]
